# Metabolomic analysis of serum may refine 21-gene expression assay risk recurrence stratification

**DOI:** 10.1038/s41523-019-0123-9

**Published:** 2019-08-29

**Authors:** Amelia McCartney, Alessia Vignoli, Leonardo Tenori, Monica Fornier, Lorenzo Rossi, Emanuela Risi, Claudio Luchinat, Laura Biganzoli, Angelo Di Leo

**Affiliations:** 1 “Sandro Pitigliani” Department of Medical Oncology, Prato Hospital, Via Suor Niccolina 20, Prato, Italy; 2 Consorzio Interuniversitario Risonanze Magnetiche di Metallo Proteine (C.I.R.M.M.P.), Via Sacconi 6, Sesto Fiorentino, 50019 Italy; 3 Centre for Magnetic Resonance (CERM), University of Florence, Via Sacconi 6, Sesto Fiorentino, 50019 Italy; 4 Department of Experimental and Clinical Medicine, University of Florence, Largo Brambilla 3, Florence, 50100 Italy; 5 Breast Medicine Service, Memorial Sloan-Kettering Cancer Center, Weill Cornell Medical College, New York, NY USA; 6 Institute of Oncology of Southern Switzerland (IOSI), Bellinzona, Switzerland; 7 Breast Unit of Southern Switzerland (CSSI), Lugano, Switzerland; 8 Department of Chemistry, University of Florence, Via della Lastruccia 3, Sesto Fiorentino, 50019 Italy

**Keywords:** Breast cancer, Prognostic markers, Cancer metabolism

## Abstract

Despite recent refinements to the 21-gene g score, allowing a better identification of patients who may derive no benefit from the addition of adjuvant chemotherapy to that of endocrine therapy, patients with early breast cancer still stand to be over-treated in the setting of clinical and/or genomic uncertainty or discordance. Here we describe and demonstrate a potential approach of further refining the OncotypeDX risk score by metabolomic analysis of serum. In a clinical dataset (*N* = 87), the risk of recurrence was further sub-stratified by metabolomic signature, with an effective splitting of each Oncotype risk classification. A total of seven recurrences were recorded, with metabolomic analysis accurately predicting six of these. Contrastingly, the genomic risk score of the seven recurrences ranged across all three Oncotype classifications (one recurrence occurred in the “low”-risk group, three in the “intermediate” group and three in the “high”-risk group).

## Introduction

In meeting its primary endpoint of distant recurrent-free survival, the recently published TAILORx study demonstrated that adjuvant endocrine therapy was non-inferior to chemotherapy plus endocrine therapy in women with endocrine receptor-positive, HER2-negative early breast cancer (eBC) whose OncotypeDX 21-gene expression assay risk recurrence scores (RS) was between 11 and 25.^1^ Nevertheless, while many cases of eBC are cured by surgery ± adjuvant endocrine therapy alone, a significant population are still over-treated due to the fear of recurrent disease established by clinicopathological and/or genomic risk factors. Genomic analysis of centrally derived tumour tissue assesses the potential, proportional benefit from adjuvant therapies, but surgery physically removes the factor (i.e. the primary tumour) upon which initial risk is estimated. Conversely, metabolomic analysis of serum detects the presence of residual micrometastatic disease, and is therefore a potential complementary tool with which to estimate residual risk of recurrence.^2–7^

Metabolomics is the -omic science that deals with the characterisation of the metabolome, in turn defined as the whole set of metabolites in a certain biological system such as a cell, a tissue, an organ or an entire organism.^8^ The two leading analytical techniques used to perform metabolomics are mass spectrometry (MS) and nuclear magnetic resonance (NMR) spectroscopy. Both techniques have their own strengths and limitations. MS overshadows NMR in terms of the number of compounds resolved, with a sensitivity in the range down to picomolar, requiring a very small volume of biospecimen. However, reproducibility remains its major limitation.^9^ Conversely, NMR analysis is high throughput and produces data that are highly reproducible and intrinsically quantitative, and thus more suitable for the fingerprinting analysis described here.^8,10^ Our group has already established a reproducible method of quantifying the individual metabolomic fingerprint, and its ability to accurately discriminate between advanced breast cancer and eBC^2^ Furthermore, we previously demonstrated that the metabolomic fingerprint can be used to predict the risk of disease recurrence in early disease,^2–4^ and that subsequent recurrence is characterised by higher (adjusted *P* < 0.05) serum levels of choline, phenylalanine, leucine, histidine, glutamate, glycine, tyrosine, valine, lactate and isoleucine.^4^

In this study, we retrospectively coupled NMR metabolomic predictions of recurrence with OncotypeDX RS in order to test the hypothesis that metabolomic prediction of risk recurrence could usefully split risk stratifications previously defined by OncotypeDX alone.

## Results

### Metabolomic analysis

**Fig. 1:**
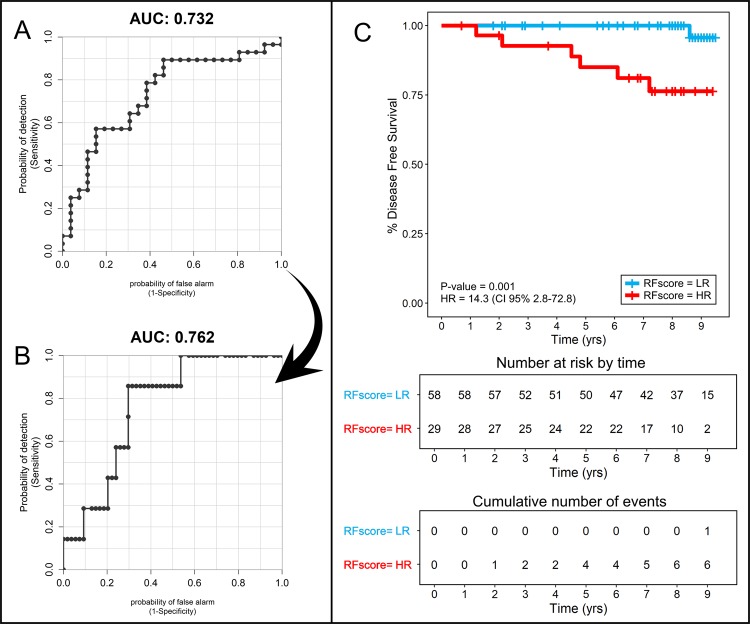
Summarised results obtained by nuclear magnetic resonance (NMR) metabolomics: **a** area under the receiver operating characteristics curve (AUC) for the Random Forest (RF) model discriminating 26 early breast cancer (eBC) patients free from cancer recurrence at follow-up and 28 metastatic breast cancer (mBC)-matched patients (training set). The score plotted is the RF risk score that expresses the probability that each sample included in the model has been classified correctly as eBC, or misclassified as mBC. **b** AUC for RF risk score of the validation set constituted by 7 eBC patients who developed recurrent disease and 54 eBC patients without recurrence. High RF risk score is deemed to represent a high risk of recurrence, as it means that the metabolomic fingerprint of an eBC patient is more closely resembles that of mBC. **c** Overall eBC patients, plotting actual disease-free survival over time (measured in years) according to estimated metabolomic risk score (Kaplan–Meier curves). “Low” (LR) and “High” (HR) RF risk patients are significantly clustered with a *P* value of 0.001 (calculated with log-rank test) and a hazard ratio of 14.3. Censored events represent either the time of last recorded clinical follow-up or time of disease recurrence. Timing of recurrent disease events is separately presented in the lower-most risk table

NMR spectra derived from the sera of 87 patients with eBC were compared with a matched population of 28 metastatic breast cancer (mBC) patients, previously analysed in a preceding study.^3^ In order to build a statistical model able to predict recurrence risk in eBC patients, 26 samples from patients with recurrence-free eBC and all mBC patients (training set) were compared using a Random Forest (RF) classifier. This model discriminated eBC with respect to mBC patients with an area under the receiver-operating characteristics (ROC) curve (AUC) of 0.732 (Fig. 1a), allowing us to obtain an RF risk score for each eBC patient. This model was tested by analysing all remaining eBC patients (validation set: 54 relapse-free patients and 7 with relapse), hypothesising that a metabolomic signature similar to that of mBC patients would be predictive of cancer recurrence. In the validation set, eBC patients without relapse were clustered with respect to relapsed patients with an AUC of 0.762 (Fig. 1b), demonstrating the predictive strength of our statistical model. Furthermore, analysing the RF risk score of all eBC (both training and validation) with Kaplan–Meier curves (Fig. 1c), a clear discrimination is evident between patients without disease recurrence and those with relapse, demonstrated by a *P* value of 0.001 and a hazard ratio (HR) of 14.3.

**Fig. 2:**
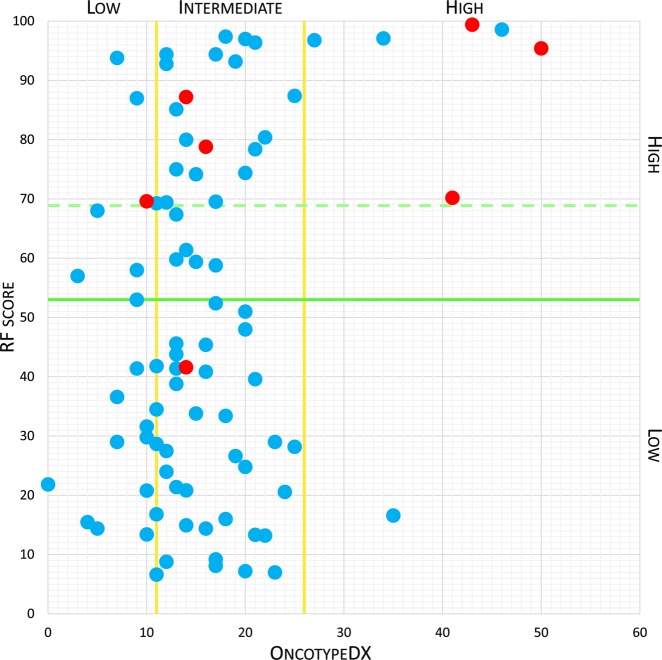
OncotypeDX score plotted against metabolomic Random Forest (RF) score. The predicted outcome based on the TAILORx-defined recurrence score classification (low/intermediate/high), sub-stratified by nuclear magnetic resonance (NMR) metabolomic RF risk score (low/high), compared to actual patient outcomes (recurrences denoted in red). The dashed line represents the cut-off for the metabolomic RF score optimised in this dataset

The metabolomic RF risk score was then combined with the predictive strength of the OncotypeDX assay, with the hypothesis that the metabolomic score could sub-classify each Oncotype-defined risk class into two subgroups: low and high risk, according to the threshold (RF ≥ 53) determined in our previous study.^3^ Furthermore, by utilising RF >69, an optimised threshold for this new dataset, improved results were obtained in the low and intermediate Oncotype risk classes (Fig. 2). In line with the landmark TAILORx study,^1^ the classification ranges of RS defined by that study are reported here.

The metabolomic RF risk score successfully split each Oncotype risk level, consequently refining genomic risk prediction. In the genomic “low-risk” group (defined by a RS ≤10), all patients with a corresponding low RF risk score were disease free at follow-up. Among the seven patients classified as high risk by RF, one relapse occurred. Within the Oncotype “intermediate-risk” class (RS 11–25), two relapses occurred in the “high-risk” RF subgroup and one in the “low-risk” subgroup. Notably, metabolic RF scores within the Oncotype “intermediate” group were heterogeneously distributed, suggesting variability in risk existing within that otherwise uniform stratification. In the “high-risk” Oncotype group (RS ≥ 26), all relapsed patients were correctly classified at “high risk” by metabolomic RF score.

## Discussion

The TAILORx trial protocol conservatively enriched one-third of its “intermediate” RS group (deemed by investigators as a RS between 11 and 25) by including 2373 patients defined as being at low risk of recurrence by classical OncotypeDX parameters (“low” risk is expressed by OncotypeDX as a RS <18). Furthermore, patients who fell into the top end of OncotypeDX-defined “intermediate-risk” group (RS 26–30) were allocated to the TAILORx-redefined “high-risk” group. It is perhaps therefore unsurprising that TAILORx found no additional benefit for chemoendocrine therapy in the revised “intermediate/mid-range” group, as a significant proportion of those assigned likely never stood to benefit from chemotherapy. An additional consequence of shifting patients with a RS between 26 and 30 to a “high-risk” group (all of whom received protocol-mandated chemoendocrine therapy) was the loss of any chance of detecting a subgroup within that classification who might safely avoid chemotherapy. Exploratory analyses of patients aged ≤50 suggested there may be benefit from chemotherapy if the RS was at the upper end of the TAILORx “mid-range” (RS 16–25), although this may be possibly attributed to the secondary ovarian suppression effected by chemotherapy in a largely pre-menopausal cohort, rather than a direct benefit of cytotoxic therapy itself. TAILORx did not identify any other factors that stratified risk of recurrence within the Oncotype-defined “intermediate-risk” group. Harnessing metabolomic analysis may potentially further this endeavour.

While exploratory in nature, this analysis represents a new integration of prognostic information derived centrally from the primary tumour, with aberrant metabolomic signalling from the periphery, which persists in the presence of micrometastatic disease. OncotypeDX RS stratifications were split further by metabolomic analysis, with all-but-one recurrences falling in “high-risk” metabolomic sets. The only patient with subsequent recurrence who was incorrectly classified by metabolomic analysis as having a “low risk” of recurrence developed cutaneous metastases ~8.5 years after initial diagnosis, on a background of having completed adjuvant anthracycline and taxane-containing chemotherapy and 5 years of endocrine therapy. This raises the question as to whether metabolomic analysis of specimens collected at the time of initial diagnosis may be limited in detecting signals of micrometastatic disease with a long lead time to clinical manifestation, or if more indolent subtypes of metastatic disease may evade metabolomic detection. The strength of this approach may be underestimated by this study, given the small number of disease recurrence events observed in the studied cohort, which was largely comprised of patients with luminal A-like disease, which often relapses beyond 5 years of completing adjuvant therapy.^11^ More relapses may yet occur in time, particularly in those patients whose metabolomic score placed them at higher risk of doing so. The results of this study require validation in a larger patient cohort.

## Methods

### Patient data and sample collection

Serum samples were selected from a breast cancer biobank belonging to Memorial Sloan Kettering Cancer Center (MSKCC), derived from patients with eBC who provided prospective written informed consent for the collection of serum and clinical information for future research purposes. Approval was obtained according to a protocol ratified by the ethics committee of MSKCC. Samples were collected post-operatively, between June 2007 and December 2009, with a mean follow-up from diagnosis of 7 years (range, 1–9 years). MSKCC maintained a database of all clinico‐pathological data and clinical outcomes, which was provided in de-identified form to the collaborating group in Italy. Study serum samples (500 μl) were maintained at −80 °C from collection until transfer over dry ice from MSKCC to Italy, where they were again stored at −80 °C until analysis. Serum samples were anonymised prior to transfer.

Of the entire MSKCC dataset (*N* = 139), all available samples from patients with early oestrogen receptor-positive/progesterone receptor-positive (PR+)/HER2-negative disease were selected for analysis. In the interests of examining as homogeneous a population as possible, samples from patients with PR-negative disease were excluded. Similarly, as OncotypeDX is not validated in patients with HER2-positive disease, HER2-positive samples were not included in this analysis. To reduce the risk of detecting a metabolomic “false-positive” signal, samples from patients who developed subsequent second primary malignancies, or a second primary breast cancer, were also excluded. The baseline characteristics of the patients (*N* = 87) included in this analysis are presented in Supplementary Table 1.^12^ Just under one-half (48%) of subjects were aged 50 years or less. Primary tumours measuring between 1.1 and 2 cm (T1c) represented 48% of the studied cohort. Although 87% of samples were from patients with node-negative disease, 80% had primary disease pathologically classed as either grade 2 or 3.

### ^1^H-NMR sample analysis

Samples were prepared following the standard protocols detailed by Bernini et al.^13^ Frozen serum samples were thawed at room temperature and shaken before use, then a total of 350 µL of a sodium phosphate buffer (10.05 g Na_2_HPO_4_·7H_2_O; 0.2 g NaN_3_; 0.4 g sodium trimethylsilyl [2,2,3,3-^2^H_4_]propionate in 500 mL of H_2_O with 20% (v/v) ^2^H_2_O; pH 7.4) was added to 350 µL of each serum sample, and the mixture was homogenised by vortexing for 30 s. A total of 600 µL of this mixture was transferred into a 5.0 mm NMR tube for the analysis.

One-dimensional ^1^H-NMR spectrum for all samples of eBC patients were acquired using a Bruker 600 MHz spectrometer (Bruker BioSpin) operating at 600.13 MHz proton Larmor frequency and equipped with a 5 mm PATXI ^1^H-, ^13^C-, ^15^N- and ^2^H-decoupling probe, including a *z*-axis gradient coil, an automatic tuning matching and an automatic and refrigerated (6 °C) sample changer (SampleJet). A BTO 2000 thermocouple served for temperature stabilisation at the level of ~0.1 K at the sample. Before measurement, samples were kept for at least 5 min inside the NMR probehead, for temperature equilibration at 310 K. Water-suppressed Carr–Purcell–Meiboom–Gill (CPMG)^14^ spin echo pulse sequence (RD-90°-(*τ*–180°-*τ*)_*n*_-acq) with a total spin echo (2*nτ*) of 80 ms was used in order to obtain one-dimensional ^1^H-NMR spectra in which broad signals from high-molecular-weight metabolites are attenuated. Eighty FIDs were collected into 73,728 data points over a spectral width of 12,019 Hz, with a relaxation delay of 4 s and acquisition time (acq) of 3.1 s. One-dimensional ^1^H-NMR CPMG spectra of serum samples from patients with metastatic hormone receptor-positive, HER2-negative breast cancer were retrieved from a pre-existing MSKCC cohort analysed in a previous study already published by our group.^3^

Free induction decays were multiplied by an exponential function equivalent to a 1.0 Hz line-broadening factor before applying Fourier transformation. Transformed spectra were automatically corrected for phase and baseline distortions and calibrated (anomeric glucose doublet at 5.24 ppm) using TopSpin 3.2 (Bruker Biospin srl).

Each 1D spectrum in the range 0.2–10.00 ppm was segmented into 0.02 ppm chemical shift bins and the corresponding spectral areas were integrated using the AMIX software (version 3.8.4, Bruker BioSpin). Binning is a means to reduce the number of total variables, to compensate for subtle signal shifts and to filter noise in the spectra, making the analysis more robust and reproducible.^15,16^ The region between 4.0 and 6.0 ppm containing the residual water signal was removed and the dimension of the system was reduced to 356 bins. Total area was used as normalisation method on the data prior to pattern recognition.

### Statistical analysis

Data analyses were performed using the open source software R. The statistical approach successfully utilised in our previous papers^3,4^ to predict the risk of disease recurrence was again applied in this study. The NMR data of the groups of 87 eBC and 28 mBC patients was randomly split into two independent cohorts: a training set consisting of 26 eBC patients recurrence free after a mean of 7 years of follow-up, and all mBC patients, plus a validation set consisting of all remaining eBC patients (54 free of recurrence and 7 with recurrent disease).

The initial analysis was restricted to the training set, with the first step to confirm that metabolomic fingerprints could distinguish between eBC patients without recurrence and mBC patients. For this purpose, a RF classifier^17^ was built. RF is a classification algorithm that uses an ensemble of unpruned decision trees (forest), each of which is built on a bootstrap sample of the training data using a randomly selected subset of variables (bins).^18,19^ The percentage of trees in the forest that assign one sample to a specific class can be inferred as a probability of belonging to a given class.^3^ In our case, each tree was used to predict whether a sample represents early or metastatic disease. For each eBC patient, a score was created that expresses the extent to which the serum metabolomic profile appeared to be similar to the profile of a confirmed metastatic sample, designated as the “RF risk score.” This score is based on the percentage of trees in the ensemble that misclassify a sample from a patient with eBC as belonging to the cohort of mBC patients. For all calculations, the R package “RF”^17^ was used to grow a forest of 500 trees, using the default settings, and ROC analysis was used to evaluate the performance of the model.

The final step was to test the hypothesis that a metabolomic signature similar to that of mBC patients would prove truly predictive of cancer recurrence. Using ROC analysis, the performance of the RF risk scores was compared with actual clinical outcome.^12^ To delineate metabolomic high risk of relapse, the cut-off for the RF score optimized in our previous study^3^ (RF ≥53) was adopted. It is worth of noting that using RF >69 improved results can be obtained in the “low” and “intermediate” Oncotype risk classes, this optimised threshold for this new dataset was determined using the function “coords” of the R package “pROC” that maximised the Youden’s *J* statistic.^20^ The ability of the RF risk score to predict breast cancer recurrences was also assessed using Kaplan–Meier curves, with additional calculation of the HR and *P* value assessed by log-rank test.

### Reporting summary

Further information on research design is available in the Nature Research Reporting Summary linked to this article.

## Supplementary information

Supplementary Table 1

NR Reporting Summary

## Data Availability

Analysed metabolomics datasets used to derive Figs 1 and 2 of the published article are publicly available in the figshare repository 10.6084/m9.figshare.8982221.^12^ Raw NMR spectra data (raw metabolomics data) supporting Figs 1 and 2 of the published article can be accessed from the corresponding author on reasonable request. Clinical (patient) data (supporting Supplementary Table 1 of the published article) are not publicly available, but can be accessed from the corresponding author on reasonable request as described at 10.6084/m9.figshare.8982221.^12^ The data generated and analysed during this study are described in the following data record: 10.6084/m9.figshare.8982221.^12^

## Supplementary information

**Supplementary information** accompanies the paper on the *npj Breast Cancer* website (10.1038/s41523-019-0123-9).
