# Supplementary material for: Metabolomic analysis of serum may refine 21-gene expression assay risk recurrence stratification
Source: NPJ Breast Cancer. 2019 Aug 29;5:26. doi: 10.1038/s41523-019-0123-9 (PMC6715716; doi:10.1038/s41523-019-0123-9)
Supplement: Supplementary file 1 — Supplementary Table 1 [file 41523_2019_123_MOESM1_ESM.pdf]

	N (%)
**Age at initial diagnosis**	
≤40	7 (8)
41-50	35 (40)
51-60	16 (18)
61-70	19 (22)
71+	10 (12)
**Primary tumour size**	
>0.1-0.5cm (T1a)	5 (6)
0.6-1cm (T1b)	25 (29)
1.1-2.0cm (T1c)	42 (48)
2.1-5.0cm (T2)	14 (16)
≥5.1cm (T3)	1 (1)
**Tumour grade**	
1	5 (6)
2	34 (39)
3	36 (41)
Unknown	12 (14)
**Nodal status**	
Positive	11 (13)
Negative	76 (87)
**Adjuvant treatment received**	
Chemotherapy	32 (37)
Radiotherapy	55 (63)
Endocrine therapy	83 (95)

Supplementary Table 1: Baseline characteristics at initial diagnosis of patients included in the early disease group metabolomic analysis. All patients had both ER and PR positive, HER2-negative disease.
